# Cancer Stem Cells Accountability in Progression of Head and Neck Squamous Cell Carcinoma: The Most Recent Trends!

**DOI:** 10.1155/2014/375325

**Published:** 2014-02-19

**Authors:** Samapika Routray, Neeta Mohanty

**Affiliations:** Department of Oral Pathology & Microbiology, Institute of Dental Sciences, SOA University, K-8, Ghatikia, Bhubaneswar, Odisha-751003, India

## Abstract

Cancer stem cells (CSCs) play a major role in local recurrence and metastatic spread in head and neck squamous cell carcinomas (HNSCC). Evidence suggests that cancer stem cells are resistant to conventional therapy. So the emerging concepts of the role of cancer stem cells in the pathobiology of HNSCC should be understood carefully to be able to create new paradigms in treatment plans.

## 1. Introduction

Despite the progress in diagnosis and advancement in management of head and neck squamous cell carcinoma (HNSCC), long-term survival rates have not improved very much over the past decade. Even after combining chemotherapy and radiation for advanced stage disease, the treatment outcomes have marginally improved [1]. The prognosis of HNSCC still remains desolate and more than 50% of patients die of this disease or complications within 5 years under current therapies [2]. Recurrence and treatment failures continue to be a major concern of HNSCC patients and late stage diagnosis accounts to be the major cause still. The initiation, growth, recurrence, and metastasis of HNSCC and other cancers have recently been related to the presence of “cancer stem cells (CSCs)” or “tumor initiating cells (TICs),” They have been demonstrated as a distinct subpopulation of tumor cells, pertaining the ability to undergo self-renewal and differentiation which further initiates the properties of normal stem cells. Hence, they possess the capacity to promote tumorigenesis and tumor growth leading to recurrence also. HNSCC appears to be supported by such cells with stem-like properties making it a significant point to be further emphasized on for eliminating the disease [3]. We, the authors, have hereby put an effort through this minireview to correlate the CSCs properties, its efficacy for tumorigenesis in HNSCC, and how to manage this entity for treatment protocols.

## 2. Cancer Stem Cells (CSCs) in relation to Tumorigenesis

### 2.1. CSCs Hypothesis in Tumorigenesis

**Figure 1:**
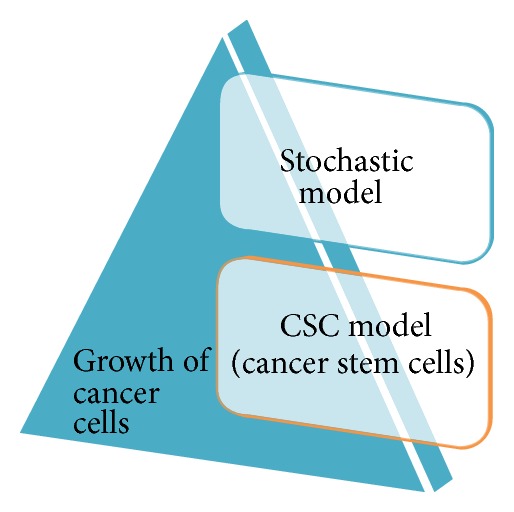


A tumor can be seen as an “organ” composed of transformed cells that interact with stromal cells within the tumor microenvironment [4]. The process of tumorigenesis requires multistep initiation of cellular and molecular pathways leading to a series of mutations resulting in the acquisition of replication and growth factor independence, resistance to growth-inhibitory signals, tissue invasion, and metastasis [5]. Does every cancer cell within the tumor having the tumor/cancer-initiating capability? Related theories suggest that there are currently two accepted models for cancer development (Figure 1) [6] as follows.

- The stochastic model suggests that every cancer cell is able to initiate new tumor growth equally.
- The alternate hypothesis is that every tumor contains a rare population of cells termed CSCs or cancer-initiating cells (CICs) [7–9].

The most recent key features of the cancer stem cell hypothesis were described by Prince and Ailles in 2008 [10]:

1. only a small fraction of the cancer cells within a tumor have tumorigenic potential when transplanted into immunodeficient mice;
2. the cancer stem cell subpopulation can be separated from the other cancer cells by distinctive surface markers;
3. tumors resulting from the cancer stem cells contain the mixed tumorigenic and nontumorigenic cells of the original tumor;
4. the cancer stem cell subpopulation can be serially transplanted through multiple generations, indicating that it is a self-renewing population.

### 2.2. Concept of CSCs

The concept of CSCs first began 150 years ago when Robert Virchow, a German pathologist, found similarities between embryonic and tumor tissues [11]. The CSC hypothesis postulates that tumor heterogeneity with regard to initiation, progression, response to therapy, and metastasis is the result of mutations which either render a normal somatic tissue stem cell cancerous or cause a cancer cell to become stem cell-like [12]. This population of tumor cells consists of rapidly dividing cells (similar to the transient amplifying (TA) cell population in normal tissue) as well as additional CSCs and more differentiated tumor cells. CSCs were first experimentally defined in hematopoietic malignancies by Lapidot and colleagues in 1994 [13]. Al-Hajj et al., in 2003, using breast tumors cells concluded that CSCs were heterogeneous in nature and a few cells isolated from primary breast cancers were capable of initiating tumors, whereas tens of thousands of phenotypically different cells did not [14].

The most common characteristics of CSCs were described as those cells having anchorage independent growth and metastasizing capacity, active membrane transporter activity, ability to survive for a long time, resistance to damaging agents, self-renewal capacity, active telomerase expression (telomere “shortening” leads to chromosomal instability), and differentiation capacity to mature progeny [15].

Gao in 2008 identified precancerous stem cells (pCSCs) in cancer and suggested that both pCSC and CSC might also serve as precursors of tumor stromal components such as tumor vasculogenic stem/progenitor cells (TVPCs). Thus, he suggested, the developing process of tumor-initiating cells (TIC) is initiated from pCSC leading to CSC and later cancer, a cellular process that parallels the histological process of hyperplasia/metaplasia (TIC) from precancerous lesions (pCSC) into malignant lesions (CSC → cancer) [16].

### 2.3. Signaling Pathways for Maintenance of CSCs

Some of the most important signals enumerated in maintaining stem cell proliferation in tumorigenesis are Oct-4, Notch, Wnt/Catenin, bone morphogenic protein (BMP), Sonic Hedgehog signalling pathway, Mushashi-1 (Msi-1), and so forth [17]. Oct-4 is normally expressed in the inner cell mass of the embryo and maintains totipotency. Notch, normally expressed in vasculature, activates endothelial cells and promotes angiogenesis. Wnt/Catenin affects orientation of chromosomes during mitotic divisions and plays a role in proliferation and inhibits apoptosis. Bone morphogenic proteins (BMP) are members of tissue growth factor-*β* (TGF-*β*) superfamily and function as oncogenes and tumour suppressors sometimes. Sonic Hedgehog signalling pathway is a major regulator of some of the fundamental processes including stem cell maintenance, cell differentiation, tissue polarity, and cell proliferation [18].

### 2.4. Methods of Identification of CSCs

CSC expresses specific markers that vary considerably depending on tumor type or tissue of origin—the discovery of a universal marker has not yet been made. General methods for the identification of CSCs in solid malignancies are similar to those strategies employed to differentiate normal stem cells from their differentiated progeny. The list of potential markers of CSCs includes CD133, CD44, CD24 (in combination with CD44) efflux of Hoechst or Rhodamine dyes, CD90, CD117, CD34, and CD20. This list also includes the efflux of vital dyes by multidrug transporters, the enzymatic activity of aldehyde dehydrogenase (ALDH), colony and sphere-forming assays utilizing specific culture conditions, and the expression of specific cell surface antigens known to enrich for stem cells [3].

The most commonly applied among them are as follows.

1. Xenotransplantation assays, the gold-standard for identification of CSCs, are used to assess the tumorigenicity and self-renewing potential of the putative CSC population.
2. CD44, the most well-recognized CSC marker, is a large cell surface glycoprotein that is involved in cell adhesion and migration.
3. ALDH (aldehyde dehydrogenase, an intracellular enzyme normally present in the liver) activity is known to enrich hematopoetic stem/progenitor cells and recently has been revealed to enrich cells with increased stem like properties in solid malignancies [19].

### 2.5. Hypoxia and CSCs

Hypoxia instigates cell demise by apoptosis/necrosis, it also prevents cell death by provoking adaptive responses that, in turn, facilitate cell proliferation or angiogenesis, causative to tumor progression [20]. In addition, studies confirm that hypoxia inhibits tumor cell differentiation and promotes maintenance of cancer stem cells and blocks the differentiation of mesenchymal stem/progenitor cells, a potential source of tumor-associated stromal cells. So an undifferentiated hypoxic microenvironment may be the initiator of cellular interactions and environmental signals for the preferential maintenance of cancer stem cells [21].

Evidence till date is suggestive of the fact that these hypoxic areas within a tumor are considered as a niche where CSCs reside. It is stated that effects of hypoxia on stem cell function are directly mediated by the hypoxia inducible factors (HIF). HIF function, as a master transcription factor, regulates hypoxia responsive genes. The molecular relationship between HIF signaling pathway with the biology of CSCs and epithelial mesenchymal transition (EMT) remains unclear although NF-*κ*B, PI3K/Akt/mTOR, Notch, Wnt/*β*-catenin, and Hedgehog signaling pathways have been recognized as important regulators of CSCs and EMT [22]. The links between the HIFs, Notch, and Oct-4 have revealed specific molecular mechanisms whereby oxygen responses can inhibit differentiation and promote stem cell identity [23]. By stimulating the expression or activity of Oct-4, Notch, and other critical signaling pathways, HIF stabilization in hypoxic tumor cells enhances stem cell properties, including self-renewal and multipotency [24].

Emerging experimental evidence indicates that hypoxia also plays an important role in the regulation of the phenotype and function of CSCs consistent with recent findings in glioma CSC. It has been shown that hypoxia is able to maintain the stem-like phenotypes in neuroblastomas and activate signaling pathways that are associated with undifferentiated phenotypes of normal stem cells, including sex determining region Y box 2 (Sox2), Oct-4, and Notch-1 signaling [25]. Both HIF-1*α* and HIF-2*α* are associated with tumor aggressiveness; HIF-1*α* promotes the phenotype and function of CSCs, whereas HIF-2*α* is highly expressed in CSCs; also known as TIC of neuroblastoma, glioblastoma, renal cell carcinoma, and breast carcinoma are associated with unfavorable disease outcome [24].

### 2.6. Metastatic Niche and CSCs

The CSC niche main role is to dedifferentiate nontumorigenic cells into tumorigenic CSCs and to induce the EMT, leading to dissemination of tumor cells from the primary tumor and further seeding at the metastatic place. Supporting cells of these niche release a number of molecular factors that have been identified to control stem cell identity. These factors include components of the BMP, Notch, Wnt, JAK-STAT, and Shh signaling pathways, which provide intercellular cues that regulate stem cell identity and differentiation [26, 27].

It has been unveiled that a premetastatic niche is established by the attraction of bone marrow derived cells to the future site of metastases by the secretion of factors from cancer cells [28]. The niche protects CSCs via differentiation and apoptosis and maintains self-regeneration via cell-cell and cell-matrix interactions which play a fundamental role in resistance to chemotherapy and radiotherapy and contribute to the genetic instability of CSCs [15]. The hypothesis of this niche, possibly for the arrival of CSCs to form a metastasis, appears to be a crucial step in metastatic spread.

## 3. CSCs Role in HNSCC Progression

### 3.1. CSCs and EMT Coexistence in HNSCC

EMT is a process by which epithelial cells lose their polarity and are converted to a migrant mesenchymal phenotype. In recent times, it has been considered as one of the fundamental steps that induces invasion and metastasis of tumors during cancer progression [29]. Similarly in HNSCC tumor microenvironment, connection between EMT and stem cell (SCs)/CSCs properties has drawn a great deal of research effort. In head and neck tumors, the overexpression of tyrosine kinase receptor B corresponds to an altered expression of the molecular mediators of EMT, including the downregulation of E-cadherin and the upregulation of Twist (a transcription factor that regulates differentiation, adhesion, and proliferation) [30]. Along with these modifications, the unique stem cell “niche” or environment is necessary to support the growth of stem cells [31].

### 3.2. CSCs Detection in HNSCC

Till date, very few studies have been conducted using HNSCC tissue for CSC marker identification. Markers such as aldehyde dehydrogenase (ALDH), CD133, and CD44 have been successfully used to identify highly tumorigenic cancer stem cells in HNSCC. Prince et al., in 2007, were the first ones to unveil the presence of highly tumorigenic, stem-like cells in HNSCC using primary tumor specimens as one-third of their samples [32]. In a follow-up study evaluating ALDH activity as a CSC marker in HNSCC, all samples were from primary tumors [33]. Later, Chen et al. showed that ALDH activity correlated with disease staging in HNSCC and that higher enzymatic activity correlated with expression of EMT genes as well as enriching cells with CSC properties [34]. In addition, ALDH activity appears to enrich CSCs in HNSCC to a higher degree than that currently provided by cell sorting based on surface antigen expression. In Table 1, we have tried to summarize the molecular markers that have been used for detection of cancer stem cells mostly in head and neck region [35–43]. Though CD44 is generally accepted as a surrogate marker for head and neck squamous carcinoma cancer stem cells (HNSC CSCs) [32], in a recent study by Oh et al., in 2013, comparing in vitro stem-like cell characteristics, chemoresistance, and in vivo tumour formation capacity of CD44+ and CD44− HNSC cells obtained from primary HNSCC patient specimens, it was suggested that CSCs themselves hold the ability to be heterogenous due to various genetic alterations and hence cannot be used as a selective marker of spheroid-forming, tumour-initiating, or chemoresistant cell populations [44]. As suggested by Zhang et al., in 2009, Hoechst 33342 dye by the ATP-binding cassette transporter (ABC) can also be used to identify side-population cells in HNSCC which have been shown to increase clonality and tumorigenic potential [45].

Lim et al. later isolated sphere-forming cells (squamospheres) from primary HNSCCs and characterized their stem cell properties like self-renewal, stem cell marker expression, aberrant differentiation, and tumor-initiating potential. Furthermore, these HNSCC-driven squamospheres appeared to be chemoresistant to cisplatin, 5-fluorouracil (FU), paclitaxel, and doxetaxel and showed increased levels of ABCG2, one of the ATP-binding cassette (ABC) transporters. So accordingly the functional analysis of these squamospheres may provide a new insight into the tumorigenic process of HNSCC [46]. But irrespective of all the above data, another study by Ishizawa et al. demonstrated a counteracting result that TICs are rare in human pancreatic adenocarcinoma, lung squamous cell carcinoma, lung adenocarcinoma, and HNSCC, despite the use of highly permissive xenotransplantation conditions [47].

## 4. CSCs Responsibility in Progression of Oral Squamous Cell Carcinoma (OSCC)

### 4.1. CSCs Link with Oral Mucosa

Oral mucosa consists of a number of distinct layers and has self-renewing capacity, such as bone marrow and skin. Mostly the basal layer cells are in the process of preparing for cell division, which later remain or move to a suprabasal position and become committed to terminal differentiation and stratification. The first hypothesis recommended that the CSCs in oral squamous cell carcinoma (OSCC) could be from local basal layer-derived adult SC or progenitor, which accumulate additional genetic alterations with time, within the tumor. The other proposed mechanism suggested the origin of CSCs to be either putative nonepithelial stem cell sources in the oral mucosa (which include vessel wall-derived cells, blood-derived stem cells, muscle-derived stem cells, and adipose-derived stem cell) or due to cell fusion between a hematopoietic stem cell and a mutated oral keratinocyte or can also originate through dedifferentiation of mature cells [48].

After understanding the possible role of CSCs in HNSCC, Oliveira et al. tried to find the possible influences of these CSCs in oral squamous cell carcinoma using CD44 and CD24. They suggested in their result that the absence of immunoexpression of these two investigated markers can be used in combination with other clinicopathologic information to improve the assessment of prognosis in OSCC [49].

### 4.2. CSCs, OSCC, EMT, and Hypoxia

Costea et al. in their hypothesis suggested a potential involvement of the stromal microenvironment OSCC progression. As already known, the activated fibroblasts (myofibroblasts) inside the tumor stroma stimulate the transformed keratinocytes, thus influencing stem cell division patterns and with further genetic alterations of these keratinocytes leads to evolution of more invasive clones [50]. OSCC is found to rely on hypoxia cellular response system for tumor progression. Focal hypoxia which is found in OSCC also may be due to quantitative and qualitative alterations in tumor vasculature, leading to local reduction of oxygen availability [51].

## 5. Therapeutic Approach

CSCs seem to harbor mechanisms that are found to be responsible for therapy resistance in glioblastoma and pancreatic cancer, and recently in colon cancer as well [52]. It has been concluded that stromal environment and CSC niche play a vital role in the behavior of cancer cells. So, targeting the stem cell niche directly can weaken the source of nutrition and change the essential signals needed by CSCs to proliferate. Therapeutic strategies as suggested by Tang et al. included targeting candidate cancer stem cells and their microenvironmental niche, which contributes to self-renewal of these cells along with the reactive oxygen species (ROS) status of these cells, and tweaking their intracellular milieu to facilitate apoptotic death signals over proliferative effects may facilitate a new prospective towards target therapy in cancers [53]. Similarly, Krishnamurthy and Nor proposed a hypothetical model for the response of HNSCC to different therapeutic strategies. As earlier established, these slow-growing cancer stem cells in HNSCC evade conventional therapies, so targeting the cancer stem cells either directly or viatheir niche could lead to a more definitive response. They suggested an emerging concept of combining the use of conventional chemotherapy and cancer stem cell-targeted therapy [4].

Hypoxia has been understood to play a key role in tumor progression and hypoxic tumor microenvironment in turn has a control over the CSCs. So, when the antiangiogenic agents are administered in combination with CSC-targeted drugs, more effective results are attained in cancer therapy, along with inhibiting hypoxia inducible factors (HIF) [54]. CSCs are less sensitive to chemo- and radiotherapy and also have a lower immunogenicity. They contribute to tumor dormancy by having a slow cell cycle kinetics (quiescent state) which protects CSCs from chemoradiotherapy [55]. Iwasaki and Suda postulated that CSCs are resistant to traditional chemotherapy, because most of the current anticancer drugs target tumor growth by inhibiting DNA synthesis or cell division of actively dividing cancer cells, as CSCs are frequently in a quiescent state [56]. So missing these quiescent CSCs can lead to resistance and relapse and may even enrich CSCs for a more resistant state.

So, the therapeutic aspect should lead to target hypoxia curbing these hypoxic niches by HIF-inhibitors, NF*κ*B-inhibitors, nutraceuticals, and antioxidant therapy. NF*κ*B degradation by inhibitor of *κ*B (I*κ*B) proteins may be a useful therapeutic solution under such circumstances. It has also been suggested that treatment with echinomycin blocks the hypoxia-induced stroma [57].

## 6. Conclusions

The confrontation in the years to come will be how to determine the contribution of each model to tumor growth in a given patient and how we can use that information to design more effective therapeutic strategy. Identification of reliable markers is required to characterize CSCs in HNSCC and OSCC in particular as this could ensure the clinical effectiveness of future targeted treatments, possibly resulting in a more effective outcome.

## Figures and Tables

**Table 1:** Molecular markers implicated in HNSCC cancer stem cell detection.

Reference	Stem cell marker	Cancer cell lines studied
Barth et al., 2004 [35]	CD34, CD117 (receptor of stem cell factor, SCF)	Oral cavity, pharynx, and larynx (stromal fibrocytes)
Kojc et al., 2005 [36]	CD34, transforming growth factor beta 1 (TGF beta 1)	Larynx
Tan et al., 2006 [37]	Stem cell factor	Nasopharynx (plasma)
Prince et al., 2007 [32]	CD44, BMI 1	HNSCC generated in immunodeficient mouse model
Zhou et al., 2007 [38]	CD133	HNSCC cell lines (hep-2)
Pries et al., 2008 [39]	CD44	Hypopharynx, larynx, and oropharynx
Chiou et al., 2008 [40]	Oct-4, Nanog, and CD133	Oral squamous cell carcinoma stem like cells
Chen et al., 2009 [41]	Bmi-1 1, Pakt (+) CD133, CD44, Nanog, and Oct-4 (−)	Tongue (SASVO3)
Chen et al., 2009 [34]	Aldehyde dehydrogenase 1 (ALDH)	Immunodeficient mouse model
Y. Wu and P. Y. Wu, 2009 [42]	CD133	Head and neck squamous cell carcinoma
Krishnamurthy et al., 2010 [43]	Aldehyde dehydrogenase (ALDH)	Head and neck squamous cell carcinoma

## References

[1] Chen Y-J, Chang JT, Liao C-T (2008). Head and neck cancer in the betel quid chewing area: recent advances in molecular carcinogenesis. *Cancer Science*.

[2] Liao C-T, Chang JT, Wang H-M (2008). Analysis of risk factors of predictive local tumor control in oral cavity cancer. *Annals of Surgical Oncology*.

[3] Wong MH, Monroe MM, Anderson EC, Clayburgh DR (2011). Cancer stem cells in head and neck squamous cell carcinoma. *Journal of Oncology*.

[4] Krishnamurthy S, Nör JE (2012). Head and neck cancer stem cells. *Journal of Dental Research*.

[5] Hanahan D, Weinberg RA (2011). Hallmarks of cancer: the next generation. *Cell*.

[6] Dalerba P, Cho RW, Clarke MF (2007). Cancer stem cells: models and concepts. *Annual Review of Medicine*.

[7] Clarke MF, Dick JE, Dirks PB (2006). Cancer stem cells—perspectives on current status and future directions: AACR workshop on cancer stem cells. *Cancer Research*.

[8] Jordan CT, Guzman ML, Noble M (2006). Cancer stem cells. *The New England Journal of Medicine*.

[9] Visvader JE, Lindeman GJ (2008). Cancer stem cells in solid tumours: accumulating evidence and unresolved questions. *Nature Reviews Cancer*.

[10] Prince MEP, Ailles LE (2008). Cancer stem cells in head and neck squamous cell cancer. *Journal of Clinical Oncology*.

[11] Huntly BJP, Gilliland DG (2005). Leukaemia stem cells and the evolution of cancer-stem-cell research. *Nature Reviews Cancer*.

[12] Zhao C, Blum J, Chen A (2007). Loss of *β*-catenin impairs the renewal of normal and CML stem cells in vivo. *Cancer Cell*.

[13] Lapidot T, Sirard C, Vormoor J (1994). A cell initiating human acute myeloid leukaemia after transplantation into SCID mice. *Nature*.

[14] Al-Hajj M, Wicha MS, Benito-Hernandez A, Morrison SJ, Clarke MF (2003). Prospective identification of tumorigenic breast cancer cells. *Proceedings of the National Academy of Sciences of the United States of America*.

[15] Allegra E, Trapasso S (2012). Cancer stem cells in head and neck cancer. *OncoTargets and Therapy*.

[16] Gao J-X (2008). Cancer stem cells: the lessons from pre-cancerous stem cells: stem cells review series. *Journal of Cellular and Molecular Medicine*.

[17] Major AG, Pitty LP, Farah CS (2013). Cancer stem cell markers in head and neck squamous cell carcinoma. *Stem Cells International*.

[18] Jena RK, Kansurkar SS, Trupti Rekha S (2012). Cancer stem cell—essence of tumorigenesis. *Journal of Carcinogenesis Mutagenesis*.

[19] Chute JP, Muramoto GG, Whitesides J (2006). Inhibition of aldehyde dehydrogenase and retinoid signaling induces the expansion of human hematopoietic stem cells. *Proceedings of the National Academy of Sciences of the United States of America*.

[20] Zhou J, Schmid T, Schnitzer S, Brüne B (2006). Tumor hypoxia and cancer progression. *Cancer Letters*.

[21] Lin Q, Yun Z (2010). Impact of the hypoxic tumor microenvironment on the regulation of cancer stem cell characteristics. *Cancer Biology and Therapy*.

[22] Bao B, Azmi AS, Ali S (2012). The biological kinship of hypoxia with CSC and EMT and their relationship with deregulated expression of miRNAs and tumor aggressiveness. *Biochimica et Biophysica Acta*.

[23] McMahon S, Charbonneau M, Grandmont S, Richard DE, Dubois CM (2006). Transforming growth factor *β*1 induces hypoxia-inducible factor-1 stabilization through selective inhibition of PHD2 expression. *The Journal of Biological Chemistry*.

[24] Keith B, Simon MC (2007). Hypoxia-inducible factors, stem cells, and cancer. *Cell*.

[25] McCord AM, Jamal M, Shankavaram UT, Lang FF, Camphausen K, Tofilon PJ (2009). Physiologic oxygen concentration enhances the stem-like properties of CD133+ human glioblastoma cells in vitro. *Molecular Cancer Research*.

[26] Joseph NM, Morrison SJ (2005). Toward an understanding of the physiological function of mammalian stem cells. *Developmental Cell*.

[27] Ohlstein B, Kai T, Decotto E, Spradling A (2004). The stem cell niche: theme and variations. *Current Opinion in Cell Biology*.

[28] Kaplan RN, Riba RD, Zacharoulis S (2005). VEGFR1-positive haematopoietic bone marrow progenitors initiate the pre-metastatic niche. *Nature*.

[29] Gupta GP, Massagué J (2006). Cancer metastasis: building a framework. *Cell*.

[30] Albers AE, Chen C, Köberle B (2012). Stem cells in squamous head and neck cancer. *Critical Reviews in Oncology/Hematology*.

[31] Li L, Xie T (2005). Stem cell niche: structure and function. *Annual Review of Cell and Developmental Biology*.

[32] Prince ME, Sivanandan R, Kaczorowski A (2007). Identification of a subpopulation of cells with cancer stem cell properties in head and neck squamous cell carcinoma. *Proceedings of the National Academy of Sciences of the United States of America*.

[33] Clay MR, Tabor M, Owen JH (2010). Single-marker identification of head and neck squamous cell carcinoma cancer stem cells with aldehyde dehydrogenase. *Head and Neck*.

[34] Chen Y-C, Chen Y-W, Hsu H-S (2009). Aldehyde dehydrogenase 1 is a putative marker for cancer stem cells in head and neck squamous cancer. *Biochemical and Biophysical Research Communications*.

[35] Barth PJ, Zu Schweinsberg TS, Ramaswamy A, Moll R (2004). CD34+ fibrocytes, *α*-smooth muscle antigen-positive myofibroblasts, and CD117 expression in the stroma of invasive squamous cell carcinomas of the oral cavity, pharynx, and larynx. *Virchows Archiv*.

[36] Kojc N, Zidar N, Vodopivec B, Gale N (2005). Expression of CD34, *α*-smooth muscle actin, and transforming growth factor *β*1 in squamous intraepithelial lesions and squamous cell carcinoma of the larynx and hypopharynx. *Human Pathology*.

[37] Tan E-L, Selvaratnam G, Kananathan R, Sam C-K (2006). Quantification of Epstein-Barr virus DNA load, interleukin-6, interleukin-10, transforming growth factor-*β*1 and stem cell factor in plasma of patients with nasopharyngeal carcinoma. *BMC Cancer*.

[38] Zhou L, Wei X, Cheng L, Tian J, Jiang JJ (2007). CD133, one of the markers of cancer stem cells in Hep-2 cell line. *Laryngoscope*.

[39] Pries R, Wittkopf N, Trenkle T, Nitsch SM, Wollenberg B (2008). Potential stem cell marker CD44 is constitutively expressed in permanent cell lines of head and neck cancer. *In Vivo*.

[40] Chiou S-H, Yu C-C, Huang C-Y (2008). Positive correlations of Oct-4 and Nanog in oral cancer stem-like cells and high-grade oral squamous cell carcinoma. *Clinical Cancer Research*.

[41] Chen C-Y, Chiou S-H, Huang C-Y (2009). Distinct population of highly malignant cells in a head and neck squamous cell carcinoma cell line established by xenograft model. *Journal of Biomedical Science*.

[42] Wu Y, Wu PY (2009). CD133 as a marker for cancer stem cells: progresses and concerns. *Stem Cells and Development*.

[43] Krishnamurthy S, Dong Z, Vodopyanov D (2010). Endothelial cell-initiated signaling promotes the survival and self-renewal of cancer stem cells. *Cancer Research*.

[44] Oh SY, Kang HJ, Kim YS, Kim H, Lim YC (2013). CD44-negative cells in head and neck squamous carcinoma also have stem-cell like traits. *European Journal of Cancer*.

[45] Zhang P, Zhang Y, Mao L, Zhang Z, Chen W (2009). Side population in oral squamous cell carcinoma possesses tumor stem cell phenotypes. *Cancer Letters*.

[46] Lim YC, Oh S-Y, Cha YY, Kim S-H, Jin X, Kim H (2011). Cancer stem cell traits in squamospheres derived from primary head and neck squamous cell carcinomas. *Oral Oncology*.

[47] Ishizawa K, Rasheed ZA, Karisch R (2010). Tumor-initiating cells are rare in many human tumors. *Cell Stem Cell*.

[48] Zhou Z-T, Jiang W-W (2008). Cancer stem cell model in oral squamous cell carcinoma. *Current Stem Cell Research and Therapy*.

[49] Oliveira LR, Oliveira-Costa JP, Araujo IM (2011). Cancer stem cell immunophenotypes in oral squamous cell carcinoma. *Journal of Oral Pathology and Medicine*.

[50] Costea DE, Tsinkalovsky O, Vintermyr OK, Johannessen AC, Mackenzie IC (2006). Cancer stem cells—new and potentially important targets for the therapy of oral squamous cell carcinoma. *Oral Diseases*.

[51] Santos d M, Mercante AMdC, Louro ID (2012). HIF1-alpha expression predicts survival of patients with squamous cell carcinoma of the oral cavity. *PLoS ONE*.

[52] Hermann PC, Bhaskar S, Cioffi M, Heeschen C (2010). Cancer stem cells in solid tumors. *Seminars in Cancer Biology*.

[53] Tang C, Ang BT, Pervaiz S (2007). Cancer stem cell: target for anti-cancer therapy. *The FASEB Journal*.

[54] Lu H, Shi S, Gong T, Zhang Z, Sun X (2013). Cancer stem cells: therapeutic implications and perspectives in cancer therapy. *Acta Pharmaceutica Sinica B*.

[55] Kusumbe AP, Bapat SA (2009). Cancer stem cells and aneuploid populations within developing tumors are the major determinants of tumor dormancy. *Cancer Research*.

[56] Iwasaki H, Suda T (2009). Cancer stem cells and their niche. *Cancer Science*.

[57] Martinez-Outschoorn UE, Trimmer C, Lin Z (2010). Autophagy in cancer associated fibroblasts promotes tumor cell survival: role of hypoxia, HIF1 induction and NF*κ*B activation in the tumor stromal microenvironment. *Cell Cycle*.

